# 同位素稀释-高分辨气相色谱/高分辨质谱测定大气中有机氯农药

**DOI:** 10.3724/SP.J.1123.2021.01001

**Published:** 2021-05-08

**Authors:** Jingxing ZHANG, Xiaoyan ZHENG, Li TAN, Jinbin LIU, Haibin YU

**Affiliations:** 中国环境监测总站, 北京 100012; China National Environmental Monitoring Centre, Beijing 100012, China; 中国环境监测总站, 北京 100012; China National Environmental Monitoring Centre, Beijing 100012, China; 中国环境监测总站, 北京 100012; China National Environmental Monitoring Centre, Beijing 100012, China; 中国环境监测总站, 北京 100012; China National Environmental Monitoring Centre, Beijing 100012, China; 中国环境监测总站, 北京 100012; China National Environmental Monitoring Centre, Beijing 100012, China

**Keywords:** 同位素稀释法, 高分辨气相色谱/高分辨质谱, 有机氯农药, 环境空气, isotope dilution (ID), high-resolution gas chromatography/high-resolution mass spectrometry (HRGC/HRMS), organochlorine pesticides, ambient air

## Abstract

建立了测定大气中25种有机氯农药(OCPs)的同位素稀释-高分辨气相色谱/高分辨质谱法(ID-HRGC/HRMS)。样品用正己烷/二氯甲烷(1:1, v/v)进行加速溶剂萃取(ASE)。通过柱洗脱实验、单柱和组合柱净化实验,最终确定样品的净化方案为弗罗里硅土固相萃取柱和石墨化炭黑固相萃取柱组合净化。样品萃取液净化后进行HRGC/HRMS分析。采用平均相对响应因子(RRF)法对样品中目标物进行定量,6点校准溶液RRF的相对标准偏差(RSD)均≤20%。线性范围为0.4~800 μg/L,相关系数*R*^2^均>0.992。对空白样品依次进行100 pg、400 pg和15 ng水平下的加标试验,各添加水平下OCPs测定值的RSD为0.64%~16%,加标回收率为67.2%~135%。穿透试验表明,滤膜+聚氨酯泡沫/聚氨酯泡沫作为吸附介质的大体积主动大气采样器(AAS)在采集环境空气时,五氯苯极易发生穿透,有效采样模式待进一步研究。在上述采样模式下,六氯苯的有效采样体积较小,标准状态(101.325 kPa, 273 K)采样体积应≤30 m^3^,其他OCPs应≤1200 m^3^。以上述体积计算,25种目标化合物的检出限为0.002~0.7 pg/m^3^。对北京环境空气样品分析测定,结果显示除反式-环氧七氯、异狄氏剂、顺式-九氯和4,4'-滴滴滴在部分样品中未检出外,其他OCPs均为100%检出;六氯苯浓度在514~563 pg/m^3^之间,其他OCPs的浓度在0.01~18.9 pg/m^3^之间;替代标回收率为33.9%~155%。由于现有相关监测标准的仪器灵敏度较低、方法检出限较高,已无法满足目前空气中痕量OCPs的测定需求,因此亟待修订新的高灵敏度监测方法标准。该方法适用于目前大气中OCPs的超痕量水平分析,为新标准的制订奠定基础,也为国家履行相关国际公约提供有力技术指导。

## Abstract (en)

A method for the determination of 25 organochlorine pesticides (OCPs) in the atmosphere using isotope dilution high-resolution gas chromatography/high-resolution mass spectrometry (ID-HRGC/HRMS) was developed. Sample extraction was performed using an accelerated solvent extractor (ASE). The extraction parameters were as follows: the extraction solvent was 50% (v/v) hexane in dichloromethane, the extraction temperature was 100 ℃, the static time was 8 min, the cell was rinsed with 60% cell volume using the aforementioned extraction solvent, the purging time was 180 s with N_2_ gas, and the extraction proceeded through three cycles. The eluting solutions of common cartridges such as florisil, graphitized carbon black, alumina, and silica were determined via cartridge elution tests. Use of the aforementioned cartridges alone cannot remove the pigments in the air sample solution. Subsequently, all possible pairwise combinations of the four cartridges were used for sample cleaning, and only the combination of florisil and graphitized carbon black was found to completely remove the pigments. Thus, the combination of florisil and graphitized carbon black cartridges using 10 mL toluene for elution was determined as the final cleaning method in this study. A high-resolution mass spectrometer equipped with a gas chromatograph was used for quantification. A fused-silica capillary column (Rtx-CL Pesticides2, 30 m×0.25 mm×0.2 μm) was used to separate the target compounds. Injection was performed in the splitless mode at 250 ℃. The flow rate of nitrogen gas was maintained constant at 1 mL/min. The oven temperature was 110 ℃ (1 min), 20 ℃/min up to 210 ℃, 1.5 ℃/min up to 218 ℃ (1 min), and 2 ℃/min up to 260 ℃ (1 min). HRMS was conducted at >8000 resolution, the source temperature was 280 ℃ in the electron impact mode using ionization energy of 35 eV, and measurements were performed in the selective ion monitoring (SIM) mode. Twenty-five OCPs were identified by comparing their GC retention times with those of the corresponding labeled compounds, and the actual ion abundance ratios of two exact *m/z* values with the corresponding theoretical values. The 25 OCPs were quantified by average relative response factors (RRFs), and the relative standard deviations (RSDs) of the RRFs with six calibration solutions were no more than 20%. The linear range of this method was 0.4 to 800 μg/L, and the correlation coefficients (*R*^2^) were higher than 0.992. To validate the method, clean materials (one quartz fiber filter (QFF) and two polyurethane foam (PUF) plugs) were spiked with 100 pg, 400 pg, and 15 ng native OCP standards, respectively; the RSDs of the 25 OCPs for each spiked level ranged from 0.64% to 16%. The spiking recoveries of the native OCPs ranged from 67.2% to 135%. Penetration experiments were conducted by sampling various volumes of air (15-1000 m^3^) using a filter-PUF/PUF high-volume active sampler. The breakthrough volume was sampled when the amount of OCPs collected in the PUF of the non-sampling end reached 5% of the total amount collected by both PUFs. When a high-volume active sampler with filter-PUF/PUF was used as an adsorbent for sampling atmospheric OCPs, a serious breakthrough of pentachlorobenzene (PeCB) occurred. The effective sampling volume of hexachlorobenzene (HCB) was very low, and was no more than 30 m^3^ under the standard conditions (101.325 kPa, 273 K). The effective sampling volumes of other OCP compounds should be no more than 1200 m^3^. This will necessitate the use of high-adsorption-capacity adsorbents such as the PUF-XAD (a styrene-divinylbenzene copolymer) sandwich used for sampling air PeCB and HCB. Calculation with the effective sampling volumes from the penetration experiment revealed that the limits of detection of the 25 OCPs were in the range of 0.002 to 0.7 pg/m^3^. Thus, the detection levels of OCPs in this study were reduced to at least 2% of the current monitoring standards. Analysis of air samples in Beijing showed that all the target compounds except for *trans*-heptachlor epoxide, endrin, *cis*-nonachlor and 4,4'-DDD were 100% detected in the air samples. The concentrations of HCB (in volumes of 15-30 m^3^) ranged from 514 to 563 pg/m^3^, while those of the other OCPs (in a volume of 600 m^3^) ranged from 0.01 to 18.9 pg/m^3^. The recoveries of surrogate standards in this sample analysis were in the range of 33.9% to 155%, which satisfied the requirements of EPA Method 1699. Because of the very high detection limits, the current related monitoring standards cannot meet the requirements of atmospheric OCP analysis, especially at the ultra-trace level. In addition, highly sensitive monitoring standard methods are urgently needed. This method is suitable for analyzing most atmospheric OCPs, even at the ultra-trace level. It also lays the foundation for a new standard method formulation and provides strong support for the implementation of relevant international conventions.

持久性有机污染物(persistent organic pollutants, POPs)是一类具有高毒性、难生物降解,能在环境中长距离迁移和扩散、可生物富集并生物放大的化合物^[1,2]^,其在各种环境介质甚至动物和人体中普遍检出^[3,4,5,6,7,8,9,10]^,在生态安全和人类健康方面产生风险,目前已引起世界范围内的广泛关注。2001年联合国环境规划署(United Nations Environment Programme, UNEP)通过了《关于持久性有机污染物的斯德哥尔摩公约》(简称公约),以消除或限制POPs生产、使用及排放,目前公约已纳入30类化合物^[11]^。

POPs具有一定挥发性,可通过大气进行扩散和迁移,因此大气中POPs的赋存水平可直观地反映环境污染的现状,UNEP全球POPs监测计划也将大气作为主要监测对象^[12]^。有机氯农药(organochlorine pesticides, OCPs)目前在公约受控清单中已多达17种^[11]^,作为典型的POPs,其大气监测分析方法的开发与优化对履约监测工作意义重大。目前相关的标准方法多为气相色谱-电子捕获检测(GC/ECD)^[13,14,15,16,17,18]^和气相色谱/质谱(GC/MS)^[19,20,21]^。这些方法的仪器分辨率较低,检出限较高,多在10^-1^~10^2^ pg/m^3[13,14,15,16,17,18,19,20]^,高于履约背景点大气中OCPs的浓度水平(一般为10^-3^~10^1^ pg/m^3^),不能满足背景点大气监测的需求^[22]^。检出限的定义和计算方式多样,不利于方法间的比较^[16-18,23-26]^。也有部分研究用到了气相色谱/串联质谱(GC/MS-MS)^[23,24,25]^和气相色谱/高分辨质谱^[26,27]^测定的方法,但是其净化方法较为简单,有待优化。同时,在大气OCPs采样方面,挥发性较强的*α*-HCH(*α*-hexachlorocyclohexane)和HCB(hexachlorobenzene)等化合物容易受环境温、湿度影响,在聚氨酯泡沫(PUF)上发生吸附穿透^[16,24,27,28]^,但很少有研究进行系统的穿透试验^[20,23-25]^,导致化合物实际污染水平被低估。

本研究采用同位素稀释-高分辨气相色谱/高分辨质谱法(ID-HRGC/HRMS)测定大气中的OCPs,在借鉴已有的ID-HRGC/HRMS测定环境样品方法的基础上^[29,30,31,32,33]^,严格根据《环境监测分析方法标准制修订技术导则》(HJ 168-2010),从大气采样、样品净化、方法适用性等多方面进行方法开发,为我国大气中的OCPs检测标准制订奠定基础,同时为履约监测提供技术支持。

## 1 实验部分

### 1.1 仪器与试剂

7890A气相色谱仪(美国Agilent公司); Autospec Premier高分辨磁质谱仪(美国Waters公司);大气主动采样器(Echo Hivol,意大利TCR TECORA公司);加速溶剂萃取仪(ASE350,美国Thermo公司)。弗罗里硅土(1 g, 6 mL)和石墨化炭黑固相萃取柱(500 mg, 6 mL, Envi-carb)(美国Supelco公司),硅胶(1 g, 6 mL)以及氧化铝固相萃取柱(1 g, 6 mL)(美国Sep-Pak公司)。

丙酮、二氯甲烷和甲苯(美国J. T. Baker公司)、正己烷(德国Merck公司)和壬烷(德国Alfa Aesar公司)均为农残级。无水硫酸钠为分析纯,使用前于400 ℃下烘烤4 h。OCPs类校准溶液(ES 5464)、天然混合标准溶液(ES 5467)、替代标溶液(ES 5465)和进样内标溶液(EC 5350)均购于美国剑桥同位素实验室。

PUF(美国Tisch公司)直径5.08 cm (2英寸),高5 cm,密度0.025 g/cm^3^,使用前用沸水烫洗后在温水中反复搓洗,沥干水分放入烘箱除水;之后用加速溶剂萃取(ASE)清洗,提取溶剂为正己烷/二氯甲烷(1∶1, v/v),于100 ℃静态平衡8 min,吹扫180 s,循环3次,冲洗比例60%;清洗完毕,置于真空干燥箱50 ℃加热8 h,密封保存。石英纤维滤膜(QFF, Munktell公司)直径102 mm,使用前于600 ℃下烘烤6 h,密封保存。

### 1.2 实验方法

1.2.1 样品采集

大流量主动采样器(active air samplers, AAS)放置于中国环境监测总站(北京)3楼楼顶采样平台,以滤膜+PUF/PUF模式采集大气中的OCPs, 220 L/min连续采样,采集约600 m^3^大气样品。采样完毕,将滤膜和PUF用铝箔包裹密封,冷藏保存直至分析。

1.2.2 样品前处理

向滤膜和PUF中加入1 ng替代标,平衡30 min后ASE提取,提取方法与PUF清洗相同。将提取液旋蒸浓缩至1~2 mL,用弗罗里硅土小柱进行净化。预先用5 mL甲苯活化小柱,上样后用10 mL甲苯洗脱并接收流出液。流出液旋蒸至约1 mL,再用石墨化炭黑小柱净化。事先用5 mL甲苯活化小柱,10 mL甲苯洗脱。流出液旋蒸、氮吹浓缩,溶剂置换为20 μL壬烷,加入1 ng进样内标,涡旋混匀后待测。

1.2.3 HRGC/HRMS条件

色谱:进样口250 ℃;载气为1.0 mL/min的高纯氦气;不分流进样,进样体积1 μL;中等极性色谱柱Rtx-CL Pesticides2(30 m×0.25 mm×0.2 μm);升温程序:110 ℃保持1 min; 20 ℃/min升温至210 ℃; 1.5 ℃/min升温至218 ℃,停留1 min; 2 ℃/min升温至260 ℃,停留1 min。

质谱:离子源温度280 ℃;电子能量35 eV;捕获电流650 μA;检测器电压350 V;动态分辨率≥8000;选择离子监测(SIM)模式,各OCPs特征离子的参数见表1。

**表 1:** 高分辨气相色谱/高分辨质谱测定OCPs的参数

No.	Compound	Retention time/min	Characteristic ion m1 (m/z)	Characteristic ion m2 (m/z)	m1/m2	
					Ratio	Tolerance/%
Target/surrogate standard						
1	hexachlorobenzene (HCB, 六氯苯)	6.85	283.8102	285.8073	1.24	±25
	^13^C_6_-HCB	6.85	289.8303	291.8273	1.24	±25
2	α-hexachlorocyclohexane (α-HCH, α-六六六)	7.10	180.9379	182.9349	1.04	±25
	^13^C_6_-α-HCH	7.08	186.9580	188.9550	1.04	±25
3	γ-HCH (γ-六六六)	7.69	180.9379	182.9349	1.04	±25
	^13^C_6_-γ-HCH	7.69	186.9580	188.9550	1.04	±25
4	β-HCH (β-六六六)	7.84	180.9379	182.9347	1.04	±25
	^13^C_6_-β-HCH	7.84	186.9580	188.9550	1.04	±25
5	δ-HCH (δ-六六六)	8.42	180.9379	182.9349	1.04	±25
	^13^C_6_-δ-HCH	8.42	186.9580	188.9550	1.04	±25
6	heptachlor (七氯)	8.53	271.8102	273.8072	1.24	±25
	^13^C_10_-heptachlor	8.53	276.8269	278.8240	1.24	±25
7	aldrin (艾氏剂)	9.26	262.8570	264.8541	1.55	±25
	^13^C_12_-aldrin	9.25	269.8804	271.8775	1.55	±25
8	oxychlordane (氧化氯丹)	10.53	386.8053	388.8024	1.05	±25
	^13^C_10_-oxychlordane	10.51	396.8387	398.8358	1.05	±25
9	cis-heptachlor epoxide (顺式-环氧七氯)	10.80	352.8442	354.8413	1.24	±25
	^13^C_10_-cis-heptachlor epoxide	10.78	362.8777	364.8748	1.24	±25
10	trans-heptachlor epoxide (反式-环氧七氯)	10.86	352.8442	354.8413	1.24	±25
	^13^C_10_-cis-heptachlor epoxide	10.78	362.8777	364.8748	1.24	±25
11	trans-chlordane (反式-氯丹)	11.39	372.8260	374.8231	1.05	±25
	^13^C_10_-trans-chlordane	11.38	382.8595	384.8565	1.05	±25
No.	Compound	Retention time/min	Characteristic ion m1 (m/z)	Characteristic ion m2 (m/z)	m1/m2	
					Ratio	Tolerance/%
12	2,4'-DDE (2,4'-滴滴伊)	11.46	246.0003	247.9975	1.56	±25
	^13^C_12_-2,4'-DDE	11.46	258.0405	260.0376	1.56	±25
13	trans-nonachlor (反式-九氯)	11.70	406.7870	408.7841	0.89	±25
	^13^C_10_-trans-nonachlor	11.70	416.8205	418.8175	0.89	±25
14	cis-chlordane (顺式-氯丹)	11.89	372.8260	374.8231	1.05	±25
	^13^C_10_-trans-chlordane	11.38	382.8595	384.8565	1.05	±25
15	endosulfan-Ⅰ (硫丹-Ⅰ)	12.06	240.9145	242.9116	0.75	±25
	^13^C_9_-endosulfan-I	12.06	248.9414	250.9384	0.75	±25
16	4,4'-DDE (4,4'-滴滴伊)	12.63	246.0003	247.9975	1.56	±25
	^13^C_12_-4,4'-DDE	12.61	258.0405	260.0376	1.56	±25
17	dieldrin (狄氏剂)	13.12	262.8570	264.8541	1.55	±25
	^13^C_12_-dieldrin	13.09	269.8804	271.8775	1.55	±25
18	2,4'-DDD (2,4'-滴滴滴)	13.43	235.0081	237.0053	1.56	±25
	^13^C_12_-2,4'-DDD	13.43	247.0483	249.0454	1.56	±25
19	endrin (异狄氏剂)	14.38	262.8570	264.8541	1.55	±25
	^13^C_12_-endrin	14.36	269.8804	271.8775	1.55	±25
20	2,4'-DDT (2,4'-滴滴涕)	14.76	235.0081	237.0053	1.55	±25
	^13^C_12_-2,4'-DDT	14.76	247.0483	249.0454	1.55	±25
21	cis-nonachlor (顺式-九氯)	14.84	406.7870	408.7841	0.89	±25
	^13^C_10_-cis-nonachlor	14.82	416.8205	418.8175	0.89	±25
22	4,4'-DDD (4,4'-滴滴滴)	15.18	235.0081	237.0053	1.56	±25
	^13^C_12_-4,4'-DDD	15.16	247.0483	249.0454	1.56	±25
23	endosulfan-Ⅱ (硫丹-Ⅱ)	15.37	240.9145	242.9116	0.75	±25
	^13^C_9_-endosulfan-Ⅱ	15.35	248.9414	250.9384	0.75	±25
24	4,4'-DDT (4,4'-滴滴涕)	16.72	235.0081	237.0053	1.55	±25
	^13^C_12_-4,4'-DDT	16.70	247.0483	249.0454	1.55	±25
25	mirex (灭蚁灵)	21.10	271.8102	273.8072	1.24	±25
	^13^C_10_-mirex	21.07	276.8269	278.8240	1.24	±25
a	pentachlorobenzene (PeCB, 五氯苯)	5.61	249.8492	251.8463	1.55	±25
	^13^C_6_-PeCB	5.61	255.8693	257.8663	1.55	±25
Injection internal standard						
b	^13^C_12_-4,4'-DiCB (^13^C_12_-4,4'-二氯联苯)	7.60	234.0406	236.0376	1.56	±25
c	^13^C_12_-2,3',4',5-TetraCB (^13^C_12_-2,3',4',5-四氯联苯)	10.69	301.9626	303.9597	0.77	±25

1.2.4 定性定量方法

在给定色谱/质谱条件下获得样品色谱/质谱峰,根据保留时间和特征离子丰度比进行定性,以平均相对响应因子(RRF)法进行定量。由校准溶液测定化合物的RRF,并计算平均值。其中23种OCPs使用其各自的^13^C标记的替代标定量;反式-环氧七氯和顺式-氯丹分别采用^13^C_10_-顺式-环氧七氯和^13^C_10_-反式-氯丹定量;所有替代标的回收率采用保留时间接近的进样内标定量。

## 2 结果与讨论

### 2.1 仪器条件的优化

比较OCPs分析常用的色谱柱DB-5MS、DB-35与专用柱Rtx-CLPesticides2对25种OCPs的分离效果,发现Rtx-CLPesticides2效果最优(见图1),且该柱对25种OCPs的仪器检出限低至其他色谱柱的5%~50%,因此选择Rtx-CLPesticides2作为分析色谱柱。

**图 1:**
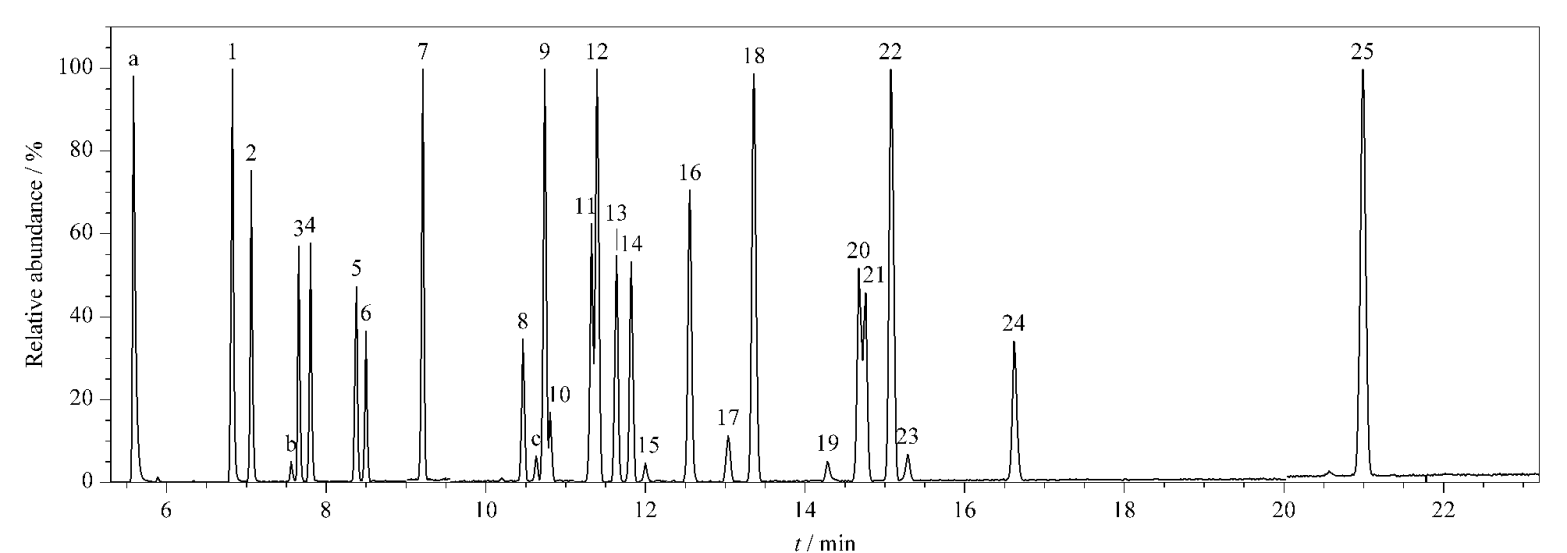
25种OCPs的总离子流图

### 2.2 净化方法优化

2.2.1 柱洗脱条件选择

调研发现,弗罗里硅土、活性炭、氧化铝以及硅胶等常用于OCPs的净化(见附表1,详见http://www.chrom-China.com/)。其中弗罗里硅土可以将OCPs与脂肪族、芳香族以及含氮化合物等干扰物相分离,石墨化炭黑能有效去除色素和甾醇类等非极性干扰物,硅胶、氧化铝可以去除有机磷酸酯和氯酚类的污染^[34]^。丙酮、二氯甲烷、甲苯、正己烷以及不同比例混合溶剂常用作上述净化柱的洗脱溶剂。

将含1 ng OCPs替代标的溶液作为模拟样品,在净化小柱活化后进行上样和洗脱,考察了不同净化方法对应的替代标回收率(见表2~表5)。

**表 2:** 弗罗里硅土小柱在不同溶剂洗脱下的替代标回收率

Compound	Recoveries/%								
	Acetone/hexane (1∶9, v/v)			Toluene			Dichloromethane/hexane (2∶8, v/v)		
10 mL	Additional 5 mL	10 mL	Additional 5 mL	10 mL	Additional 5 mL	Additional 10 mL dichloromethane/hexane (3∶7, v/v)
^13^C_6_-PeCB	39	0		75	0		28	0	0
^13^C_6_-HCB	34	0		61	0		30	0	0
^13^C_6_-α-HCH	53	0		75	0		43	0	0
^13^C_6_-γ-HCH	57	0		70	0		46	0	0
^13^C_6_-β-HCH	58	0		53	0		52	0	0
^13^C_6_-δ-HCH	67	0		84	0		68	0	0
^13^C_10_-Heptachlor	56	0		60	0		45	0	0
^13^C_12_-Aldrin	59	0		84	0		49	0	0
^13^C_10_-Oxychlordane	60	0		83	0		55	0	0
^13^C_10_-cis-Heptachlor epoxide	65	0		78	0		51	5	1
^13^C_10_-trans-Chlordane	54	0		64	0		50	0	0
^13^C_12_-2,4'-DDE	77	0		98	0		73	0	0
^13^C_10_-trans-Nonachlor	49	0		47	0		47	0	0
^13^C_9_-Endosulfan-Ⅰ	65	0		80	0		5	12	53
^13^C_12_-4,4'-DDE	78	0		92	0		75	0	0
^13^C_12_-Dieldrin	67	0		100	0		1	8	60
^13^C_12_-2,4'-DDD	89	0		91	0		89	0	0
^13^C_12_-Endrin	82	0		83	0		2	5	70
^13^C_12_-2,4'-DDT	91	0		61	0		88	0	0
^13^C_10_-cis-Nonachlor	56	0		41	0		54	0	0
^13^C_12_-4,4'-DDD	94	0		86	0		95	0	0
^13^C_9_-Endosulfan-Ⅱ	70	0		79	0		0	0	7
^13^C_12_-4,4'-DDT	98	0		51	0		93	0	0
^13^C_10_-Mirex	80	0		76	0		77	0	0

**表 3:** 石墨化炭黑小柱在不同溶剂洗脱下的替代标回收率

Compound	Recoveries/%							
	Toluene			Acetone/hexane (1∶1, v/v)			Dichloromethane/hexane (1∶1, v/v)	
10 mL	Additional 5 mL	10 mL	Additional 5 mL	10 mL	Additional 5 mL
^13^C_6_-PeCB	61	1		0	0		13	2
^13^C_6_-HCB	104	1		0	0		0	0
^13^C_6_-α-HCH	56	1		45	0		47	0
^13^C_6_-γ-HCH	55	1		54	0		52	1
^13^C_6_-β-HCH	49	1		66	0		61	1
^13^C_6_-δ-HCH	55	1		80	0		76	1
^13^C_10_-Heptachlor	83	1		62	0		59	1
^13^C_12_-Aldrin	83	1		63	0		62	0
^13^C_10_-Oxychlordane	102	1		75	0		79	1
^13^C_10_-cis-Heptachlorepoxide	79	1		79	0		81	1
^13^C_10_-trans-Chlordane	113	1		77	0		79	1
^13^C_12_-2,4'-DDE	87	1		93	0		97	1
^13^C_10_-trans-Nonachlor	150	1		73	0		72	1
^13^C_9_-Endosulfan-Ⅰ	67	1		84	0		91	1
^13^C_12_-4,4'-DDE	74	1		97	0		102	1
^13^C_12_-Dieldrin	80	1		86	0		94	1
^13^C_12_-2,4'-DDD	87	1		105	0		114	2
^13^C_12_-Endrin	79	0		103	0		102	1
^13^C_12_-2,4'-DDT	77	1		99	0		103	1
^13^C_10_-cis-Nonachlor	133	1		73	0		76	1
^13^C_12_-4,4'-DDD	81	1		110	0		119	2
^13^C_9_-Endosulfan-Ⅱ	74	1		96	1		101	1
^13^C_12_-4,4'-DDT	80	1		108	0		110	1
^13^C_10_-Mirex	72	1		88	0		94	1

**表 4:** 氧化铝小柱在不同溶剂洗脱下的替代标回收率

Compound	Recoveries/%				
	Dichloromethane/hexane (1∶9, v/v)			Hexane	
	10 mL	Additional 5 mL		10 mL	Additional 5 mL dichloromethane/hexane (2∶8, v/v)
^13^C_6_-PeCB	25	0		28	0
^13^C_6_-HCB	30	0		31	0
^13^C_6_-α-HCH	40	0		41	0
^13^C_6_-γ-HCH	42	0		43	0
^13^C_6_-β-HCH	50	0		35	12
^13^C_6_-δ-HCH	58	3		0	66
^13^C_10_-Heptachlor	50	0		48	0
^13^C_12_-Aldrin	56	0		56	0
^13^C_10_-Oxychlordane	62	0		56	0
^13^C_10_-cis-Heptachlor epoxide	65	0		61	0
^13^C_10_-trans-Chlordane	55	0		50	0
^13^C_12_-2,4'-DDE	78	0		71	0
^13^C_10_-trans-Nonachlor	51	0		45	0
^13^C_9_-Endosulfan-Ⅰ	65	0		59	1
^13^C_12_-4,4'-DDE	82	0		73	0
^13^C_12_-Dieldrin	74	0		69	0
^13^C_12_-2,4'-DDD	93	0		84	0
^13^C_12_-Endrin	84	0		68	0
^13^C_12_-2,4'-DDT	97	0		85	0
^13^C_10_-cis-Nonachlor	60	0		53	0
^13^C_12_-4,4'-DDD	102	0		89	0
^13^C_9_-Endosulfan-Ⅱ	55	14		1	71
^13^C_12_-4,4'-DDT	104	0		87	0
^13^C_10_-Mirex	83	0		75	0

**表 5:** 硅胶小柱在不同溶剂洗脱下的替代标回收率

Compound	Recoveries/%				
	Dichloromethane/hexane (1∶1, v/v)			Hexane	
10 mL	Additional 5 mL	10 mL	Additional 5 mL dichloromethane
^13^C_6_-PeCB	33	0		29	0
^13^C_6_-HCB	46	0		38	0
^13^C_6_-α-HCH	46	0		42	1
^13^C_6_-γ-HCH	43	0		47	1
^13^C_6_-β-HCH	50	1		6	60
^13^C_6_-δ-HCH	60	1		0	76
^13^C_10_-Heptachlor	62	0		55	0
^13^C_12_-Aldrin	65	0		60	0
^13^C_10_-Oxychlordane	70	0		74	1
^13^C_10_-cis-Heptachlor epoxide	69	1		49	28
^13^C_10_-trans-Chlordane	72	0		78	1
^13^C_12_-2,4'-DDE	79	1		86	1
^13^C_10_-trans-Nonachlor	67	1		74	1
^13^C_9_-Endosulfan-Ⅰ	71	1		66	5
^13^C_12_-4,4'-DDE	80	1		91	0
^13^C_12_-Dieldrin	73	1		36	47
^13^C_12_-2,4'-DDD	84	1		94	1
^13^C_12_-Endrin	67	1		54	28
^13^C_12_-2,4'-DDT	84	0		90	0
^13^C_10_-cis-Nonachlor	67	1		63	14
^13^C_12_-4,4'-DDD	84	1		97	2
^13^C_9_-Endosulfan-Ⅱ	73	1		0	76
^13^C_12_-4,4'-DDT	83	0		93	1
^13^C_10_-Mirex	71	0		85	0

弗罗里硅土小柱的洗脱结果(见表2)表明,丙酮/正己烷(1∶9, v/v)以及甲苯作洗脱溶剂时,洗脱效果均较好,且从追加5 mL洗脱溶剂的结果来看,10 mL溶剂用量已足够。二氯甲烷/正己烷(2∶8, v/v)对硫丹-Ⅰ、狄氏剂、异狄氏剂和硫丹-Ⅱ的洗脱效果较差,追加的10 mL二氯甲烷/正己烷(3∶7, v/v)虽能洗脱50%~70%的硫丹-Ⅰ、狄氏剂和异狄氏剂,但硫丹-Ⅱ的回收率仅达7%,洗脱效果仍不尽人意。整体而言10 mL甲苯的洗脱效果最突出,因此后续实验中此柱的洗脱溶剂定为10 mL甲苯。

石墨化炭黑小柱的洗脱结果(见表3)表明,甲苯的洗脱效果较好,且10 mL用量已足够。丙酮/正己烷(1∶1, v/v)和二氯甲烷/正己烷(1∶1, v/v)不能有效地将六氯苯从柱上洗脱,故后续实验选择10 mL甲苯作为该柱的洗脱溶剂。

氧化铝小柱的洗脱结果(见表4)表明,二氯甲烷/正己烷(1∶9, v/v)作洗脱溶剂时,六氯苯的回收率约为30%, *α*-HCH和*γ*-HCH的回收率约为40%,其他均>50%。10 mL正己烷作洗脱溶剂时,不能将硫丹-Ⅱ和*δ*-HCH从柱上洗脱,可能是正己烷极性太弱所致,而追加的5 mL二氯甲烷/正己烷(2∶8, v/v)洗脱溶剂下*δ*-HCH和硫丹-Ⅱ的回收率高达60%~80%。考虑到整体洗脱效果和简化操作,后续实验选择15 mL二氯甲烷/正己烷(1∶9, v/v)作为氧化铝小柱的洗脱溶剂。

硅胶小柱的洗脱结果(见表5)表明,二氯甲烷/正己烷(1∶1, v/v)的洗脱效果较好,化合物回收率均在46%以上(五氯苯的回收率为33%),且10 mL用量已足够。与氧化铝小柱相似,正己烷也不能有效地将硫丹-Ⅱ和*δ*-HCH从硅胶柱上洗脱,且*β*-HCH的回收率仅为6%。追加5 mL二氯甲烷后,狄氏剂、异狄氏剂回收率增加,*β*-HCH、*δ*-HCH和硫丹-Ⅱ的回收率大大增加。考虑整体洗脱效果和简化操作,后续实验选择10 mL二氯甲烷/正己烷(1∶1, v/v)作为此柱的洗脱溶剂。

2.2.2 单一填料柱净化

多个空气样品经过提取后,合并提取液,浓缩并定容至10 mL,制备统一样品溶液。取0.5 mL(*n*=2),加入1 ng替代标,混匀后分别用弗罗里硅土、石墨化炭黑、氧化铝和硅胶小柱进行净化,各净化柱均用上文中的较优洗脱溶液进行洗脱,将流出液收集后浓缩至20 μL,结果发现浓缩液色素均较重,即单一净化柱不能有效去除样品溶液色素,为减少色素对仪器测样干扰,本文进一步研究了组合净化柱。

2.2.3 组合填料柱净化

将统一样品溶液分别进行组合净化,组合方式及净化效果见表6。弗罗里硅土小柱和石墨化炭黑小柱组合能够很好地去除样品色素,减弱对目标物出峰的干扰,延长色谱柱寿命。进一步将经过弗罗里硅土小柱和石墨化炭黑小柱组合净化的大气样品溶液进行仪器测定,样品中OCPs的替代标回收率为33.9%~155%。

**表 6:** 组合固相萃取小柱对空气样品中OCPs的净化效果

Cartridge combination	Elution solvent	Pigments remain
Silica→florisil	silica: 10 mL dichloromethane/hexane (1∶1, v/v); florisil: 10 mL toluene	yes
Silica→graphitized carbon black	silica: 10 mL dichloromethane/hexane (1∶1, v/v); graphitized carbon black:	yes
	10 mL toluene	
Silica→alumina	silica: 10 mL dichloromethane/hexane (1∶1, v/v); alumina: 15 mL	yes
	dichloromethane/hexane (1∶9, v/v)	
Florisil→alumina	florisil: 10 mL toluene; alumina: 15 mL dichloromethane/hexane (1∶9, v/v)	yes
Florisil→graphitized carbon black	florisil: 10 mL toluene; graphitized carbon black: 10 mL toluene	no
Alumina→graphitized carbon black	alumina: 15 mL dichloromethane/hexane (1∶9, v/v); graphitized carbon black:	yes
	10 mL toluene	

### 2.3 方法适用性

2.3.1 穿透试验

AAS因能通过采样泵加流量计精确控制大气采样量,而广泛应用于大气OCPs采样。AAS吸附介质多为滤膜+PUF模式,但是一些OCPs挥发性较强,采样时容易在PUF上发生穿透,所以现场采样前应先确定有效采样体积。确定方式主要有以下两种^[14]^:一种是穿透试验,即串联2块及以上PUF进行采样,计算下层PUF吸附目标物量相对上层PUF或总PUF的比值^[16,27,28]^;另一种是动态保留试验,即向PUF气体流入端加标,采样后计算加标回收率^[15,19,35]^。其中穿透试验的方法在研究中更常见,但穿透标准不一,如穿透限值存在下层PUF占上层PUF的33.3%^[28]^或50%^[27]^、下层PUF占总PUF比值5%^[36]^等多种说法(见附表1,详见http://www.chrom-China.com/)。鉴于PUF对HCB和*α*-HCH的吸附容量极易受环境温、湿度影响^[16,27]^,本文采用穿透试验的方式,并选择定义下层PUF吸附目标物量占总PUF比值(简称“穿透比率”)5%即为穿透这一严格标准,以增强本次实验结果的指导意义。2019年2月,在中国环境监测总站(北京)3楼楼顶进行穿透试验,以滤膜+PUF/PUF模式,多台主动采样器同时采集不同体积的空气样品,各体积采集1个平行样。采样结束后,分别测定两层PUF中OCPs的含量,计算下层PUF吸附的目标物占总PUF吸附量的比值,结果见表7(采样体积已换算为标准状态(101.325 kPa, 273 K)对应的体积)。

**表 7:** 穿透实验中下层PUF吸附目标物占总PUF吸附量的比值

Compound	Ratios at different sampling volumes/%																									
	15 m^3^		30 m^3^		53 m^3^		181 m^3^		271 m^3^		387 m^3^		486 m^3^		607 m^3^		677 m^3^		780 m^3^		892 m^3^			958 m^3^		
PeCB	43		48		53		49		53		51		59		58		50		41		36			35		
HCB	5	.0	25		37		45		43		49		24		30		41		55		61			59		
α-HCH	N	.D.	1	.0	13		3	.0	4	.4	13		0	.5	0	.9	4	.3	10		28			20		
γ-HCH	N	.D.	N	.D.	N	.D.	N	.D.	N	.D.	N	.D.	1	.1	1	.3	6	.8	0	.8	2	.8		3		.4
β-HCH	N	.D.	N	.D.	N	.D.	N	.D.	N	.D.	N	.D.	N	.D.	N	.D.	N	.D.	N	.D.	1	.1		3		.5
δ-HCH	N	.D.	N	.D.	N	.D.	N	.D.	N	.D.	N	.D.	N	.D.	N	.D.	N	.D.	N	.D.	0	.8		0		.9
Heptachlor	5	.0	N	.D.	N	.D.	N	.D.	N	.D.	N	.D.	0	.6	0	.9	7	.0	0	.4	7	.2		1		.8
Aldrin	2	.2	1	.6	1	.1	1	.0	1	.5	2	.8	1	.6	1	.6	3	.8	1	.3	1	.6		1		.9
Oxychlordane	N	.D.	N	.D.	N	.D.	N	.D.	N	.D.	N	.D.	4	.4	4	.3	3	.6	3	.5	2	.0		2		.5
cis-Heptachlor epoxide	N	.D.	N	.D.	N	.D.	N	.D.	N	.D.	N	.D.	2	.4	2	.3	2	.2	2	.1	1	.2		1		.5
trans-Heptachlor epoxide	N	.D.	N	.D.	N	.D.	N	.D.	N	.D.	N	.D.	N	.D.	N	.D.	N	.D.	N	.D.	N	.D.		N		.D.
trans-Chlordane	N	.D.	1	.5	1	.5	3	.4	2	.7	2	.0	4	.2	4	.4	5	.3	2	.5	1	.4		2		.5
2,4'-DDE	N	.D.	N	.D.	N	.D.	N	.D.	N	.D.	N	.D.	2	.0	3	.2	2	.1	1	.2	1	.0		0		.9
trans-Nonachlor	1	.6	0	.9	1	.7	2	.9	1	.3	1	.2	1	.8	2	.1	2	.8	1	.6	0	.2		1		.2
cis-Chlordane	N	.D.	N	.D.	N	.D.	2	.4	2	.7	1	.5	2	.7	1	.0	3	.5	0	.9	0	.5		1		.5
Endosulfan-Ⅰ	1	.7	0	.6	N	.D.	0	.2	0	.3	0	.2	5	.4	5	.1	6	.7	3	.9	0	.4		1		.5
4,4'-DDE	N	.D.	N	.D.	N	.D.	0	.3	N	.D.	0	.2	1	.6	1	.4	1	.7	0	.3	0	.2		0		.2
Dieldrin	N	.D.	N	.D.	N	.D.	3	.4	3	.7	2	.1	2	.9	2	.4	2	.8	5	.4	1	.2		2		.7
2,4'-DDD	N	.D.	N	.D.	N	.D.	N	.D.	N	.D.	N	.D.	N	.D.	N	.D.	N	.D.	8	.4	3	.6		5		.0
Endrin	N	.D.	N	.D.	N	.D.	N	.D.	N	.D.	N	.D.	N	.D.	N	.D.	N	.D.	N	.D.	8	.4		7		.5
2,4'-DDT	N	.D.	N	.D.	N	.D.	N	.D.	N	.D.	N	.D.	N	.D.	2	.8	3	.3	1	.5	0	.9		1		.0
cis-Nonachlor	N	.D.	N	.D.	N	.D.	N	.D.	N	.D.	N	.D.	N	.D.	N	.D.	N	.D.	N	.D.	N	.D.		N		.D.
4,4'-DDD	N	.D.	N	.D.	N	.D.	N	.D.	N	.D.	N	.D.	N	.D.	N	.D.	N	.D.	N	.D.	4	.7		6		.7
Endosulfan-Ⅱ	N	.D.	9	.5	N	.D.	0	.7	2	.3	0	.9	0	.0	5	.3	N	.D.	N	.D.	N	.D.		N		.D.
4,4'-DDT	N	.D.	N	.D.	N	.D.	N	.D.	N	.D.	N	.D.	N	.D.	N	.D.	N	.D.	2	.4	1	.0		1		.3
Mirex	2	.9	1	.7	1	.5	0	.4	0	.6	0	.5	0	.8	1	.5	2	.2	0	.6	0	.3		0		.5

N.D.: not detected in the lower layer.

由表7可见,样品中下层PUF吸附的五氯苯和六氯苯的量在总PUF中占比均较大。其中五氯苯在采样体积为15 m^3^时,穿透比率也已超出40%,即严重穿透;六氯苯在低于50 m^3^时,穿透比率较低,其中在15 m^3^时为5%; *α*-六六六在53和387 m^3^时,穿透比率为13%,在采样体积≤677 m^3^时,穿透比率均<5%,可能是期间天气变化较大所致;其他OCPs在采样体积为607 m^3^时,整体而言穿透比率均在5%左右。

综上,五氯苯不适合滤膜+PUF/PUF的采样模式,建议与XAD(苯乙烯-二乙烯基苯共聚物)或Tenax-TA(聚2,6-二苯基对苯醚)等强吸附性能介质相结合。对于*α*-HCH,采样时应尽量避开高湿度天气。在相似采样环境下安装单块PUF,六氯苯的采样体积应≤15 m^3^,双块PUF时应≤30 m^3^。其他OCPs采样体积为,单块PUF应≤600 m^3^,双块PUF应≤1200 m^3^。

2.3.2 平均相对响应因子、线性范围、相关系数和检出限

测定0.4~800 μg/L的OCPs校准溶液,计算目标物和替代标的平均相对响应因子,其对应的RSD≤20%,线性相关系数(*R*^2^)均>0.992,详见表8。按照HJ 168要求,计算方法的检出限(MDL):按照样品分析的全流程重复测定7次空白试样,测定结果以浓度表示,其中六氯苯按采样体积30 m^3^计算,其他24种OCPs按1200 m^3^计算,得出测定结果的标准偏差(*S*), MDL为3.143*S*,对空白试验中未检出的目标物进行加标后测定,判断各目标物MDL的合理性,必要时调整加标量。六氯苯的MDL为0.7 pg/m^3^,其他24种OCPs的MDL为0.002~0.007 pg/m^3^,详见表8。

**表 8:** 25种OCPs的方法检出限、线性相关系数和加标回收率

Compound	MDL/ (pg/m^3^)	R^2^	Recovery/ %
HCB	0.7	0.9986	90.2-125
α-HCH	0.006	0.9997	88.6-135
γ-HCH	0.007	0.9998	92.9-121
β-HCH	0.004	0.9999	97.8-117
δ-HCH	0.004	0.9999	92.0-110
Heptachlor	0.003	0.9994	77.6-102
Aldrin	0.006	1.0000	82.1-98.6
Oxychlordane	0.003	0.9996	81.4-124
cis-Heptachlor epoxide	0.002	0.9999	83.3-103
trans-Heptachlor epoxide	0.002	0.9969	70.9-104
trans-Chlordane	0.006	0.9994	76.8-120
2,4'-DDE	0.003	0.9999	76.0-102
trans-Nonachlor	0.003	0.9998	76.5-130
cis-Chlordane	0.004	0.9987	76.2-118
Endosulfan-Ⅰ	0.004	0.9929	67.2-120
4,4'-DDE	0.004	0.9999	80.0-102
Dieldrin	0.003	0.9997	82.4-104
2,4'-DDD	0.003	0.9999	74.9-98.4
Endrin	0.003	0.9990	75.7-107
2,4'-DDT	0.003	0.9995	82.6-108
cis-Nonachlor	0.002	0.9993	75.9-130
4,4'-DDD	0.003	0.9998	84.8-98.2
Endosulfan-Ⅱ	0.005	0.9973	81.5-115
4,4'-DDT	0.003	0.9990	82.2-99.3
Mirex	0.003	0.9999	85.6-101

2.3.3 精密度和回收率

向空白滤膜和PUF中加入低(100 pg)、中(400 pg)、高(15 ng)3个水平的OCPs标准物质(*n*=6),按照实际样品处理流程进行提取、净化和仪器分析,计算同一加标水平下OCPs测定值的RSD。3个加标水平下,测定值的RSD均在0.64%~16%之间,加标回收率为67.2%~135%,详见表8。

### 2.4 空气样品的测定结果

根据穿透情况,采集15~30 m^3^的空气样品,测得六氯苯的浓度为514~563 pg/m^3^。采集约600 m^3^的空气样品,测得除反式-环氧七氯、异狄氏剂、顺式-九氯和4,4'-滴滴滴在部分样品中未检出外,其他OCPs均为100%检出,浓度为0.01~18.9 pg/m^3^,低于现有标准HJ 900的检出限(0.03~0.07 ng/m^3^),所有样品的替代标回收率为33.9%~155%。

## 3 结论

本文建立了弗罗里硅土小柱与石墨化炭黑小柱组合净化,中等极性Rtx-CL Pesticides2色谱柱分离,同位素稀释-HRGC-HRMS分析大气25种OCPs的方法。空白样品进行低、中和高3个水平的加标试验,测定结果的RSD为0.64%~16%,加标回收率为67.2%~135%。实际样品分析中替代标的回收率为33.9%~155%。穿透试验确定六氯苯的有效采样体积(标态)应≤30 m^3^,其他24种OCPs应≤1200 m^3^。以上述体积计算,方法的检出限为0.002~0.7 pg/m^3^。该方法较系统、全面,测定干扰因素较少,回收率较好,检出限能很好地满足当前大气中痕量OCPs的测定需求,可用于履约监测以及大规模大气样品的调查。
